# Obesity Alters the Muscle Protein Synthetic Response to Nutrition and Exercise

**DOI:** 10.3389/fnut.2019.00087

**Published:** 2019-06-13

**Authors:** Joseph W. Beals, Nicholas A. Burd, Daniel R. Moore, Stephan van Vliet

**Affiliations:** ^1^Center for Human Nutrition, Washington University School of Medicine, St. Louis, MO, United States; ^2^Department of Kinesiology and Community Health, University of Illinois at Urbana-Champaign, Urbana, IL, United States; ^3^Faculty of Kinesiology and Physical Education, University of Toronto, Toronto, ON, Canada; ^4^Duke Molecular Physiology Institute, Duke University Medical Center, Durham, NC, United States

**Keywords:** exercise, fat mass, muscle mass, mTORC1, p70S6K, inflammation, TLR4, anabolic resistance

## Abstract

Improving the health of skeletal muscle is an important component of obesity treatment. Apart from allowing for physical activity, skeletal muscle tissue is fundamental for the regulation of postprandial macronutrient metabolism, a time period that represents when metabolic derangements are most often observed in adults with obesity. In order for skeletal muscle to retain its capacity for physical activity and macronutrient metabolism, its protein quantity and composition must be maintained through the efficient degradation and resynthesis for proper tissue homeostasis. Life-style behaviors such as increasing physical activity and higher protein diets are front-line treatment strategies to enhance muscle protein remodeling by primarily stimulating protein synthesis rates. However, the muscle of individuals with obesity appears to be resistant to the anabolic action of targeted exercise regimes and protein ingestion when compared to normal-weight adults. This indicates impaired muscle protein remodeling in response to the main anabolic stimuli to human skeletal muscle tissue is contributing to poor muscle health with obesity. Deranged anabolic signaling related to insulin resistance, lipid accumulation, and/or systemic/muscle inflammation are likely at the root of the anabolic resistance of muscle protein synthesis rates with obesity. The purpose of this review is to discuss the impact of protein ingestion and exercise on muscle protein remodeling in people with obesity, and the potential mechanisms underlining anabolic resistance of their muscle.

## Introduction

At present, 39% of adult Americans are obese (1), which is defined as having a body mass index (BMI) of 30 or higher. Obesity represents a growing societal problem as incidence has increased rapidly since the early 2000s when 30% of American were obese (2). If current trends continue, it is projected that nearly half of all US adults may be obese by 2030 (3). Obesity is associated with several chronic conditions including cancer, type 2 diabetes, cardiovascular disease, arthritis, liver and kidney disease, sleep apnea, mental illness (4, 5), and increased risk of all-cause mortality (6). The annual obesity-related healthcare costs in 2005 were estimated at $190.2 billion or nearly 21% of total medical spending in the US (7). Therefore, effective treatment strategies to prevent, halt, and reverse obesity are imperative to improve public health and reduce the societal (e.g., healthcare and economic) cost of obesity.

The frontline treatment of obesity is typically multifactorial and is predominantly centered around behavior strategies such as changes in nutrition and/or physical activity to elicit weight loss (8). Weight loss, however, commonly occurs with concomitant reductions in skeletal muscle mass (9), the prevention of which is a focus for several research groups (10, 11). These efforts are due to the recognized important contribution of skeletal muscle health to total body health (12).

**Figure 1:**
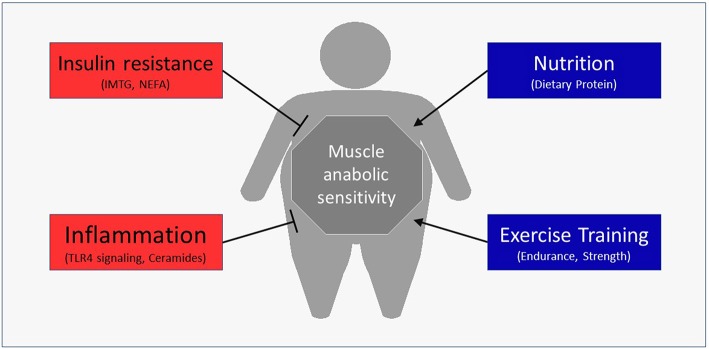
Potential regulators of the postprandial muscle protein synthetic response with obesity.

Besides the obvious role in generating force for movement, skeletal muscle also contributes to health through the use and storage of macronutrients (13). Skeletal muscle is the primary determinant of meal-derived glucose (14) and lipid (15) uptake and utilizes a major portion of meal-derived amino acids (AA) released into systemic circulation to build new functional proteins (16, 17). Moreover, changes in the skeletal muscle's contribution to basal and/or postprandial macronutrient metabolism can have profound effects on disease risk (18). For example, impaired insulin sensitivity is a fundamental characteristic of Type 2 diabetes (19). Emerging data have revealed that obesity may also negatively alter muscle protein turnover, or the breaking down and rebuilding of functional proteins, with the myofibrillar proteins being particularly susceptible to anabolic resistance. The purpose of this review is to discuss the mechanisms by which obesity may hamper normal turnover of muscle proteins and ultimately impact muscle health (Figure 1). In addition, we discuss lifestyle strategies to improve the muscle protein synthetic response with obesity.

## Skeletal Muscle Protein Turnover for Healthy Muscle

Tissue proteins are maintained through the coordination of rates of synthesis (from free AAs) and breakdown (replenishment of the free amino acid pool) under basal conditions. In skeletal muscle, protein synthesis appears to be more highly responsive to changes in plasma amino acid (AA) availability as compared to protein breakdown (MPB) (20–22). Proteins in skeletal muscle are degraded for a variety of reasons including to remodel the muscle in response to changes in metabolic demands (e.g., larger and stronger vs. more fatigue resistant muscles) (23, 24) or as they become old, damaged and subsequently need replacement (25–27). Moreover, as the body's largest pool of AAs, the muscle provides gluconeogenic precursors to other tissues during an overnight (28, 29) or prolonged fast (30). Therefore, the stimulation of muscle protein synthesis rates represents an important physiological process for maintaining the health and function of this tissue.

**Figure 2:**
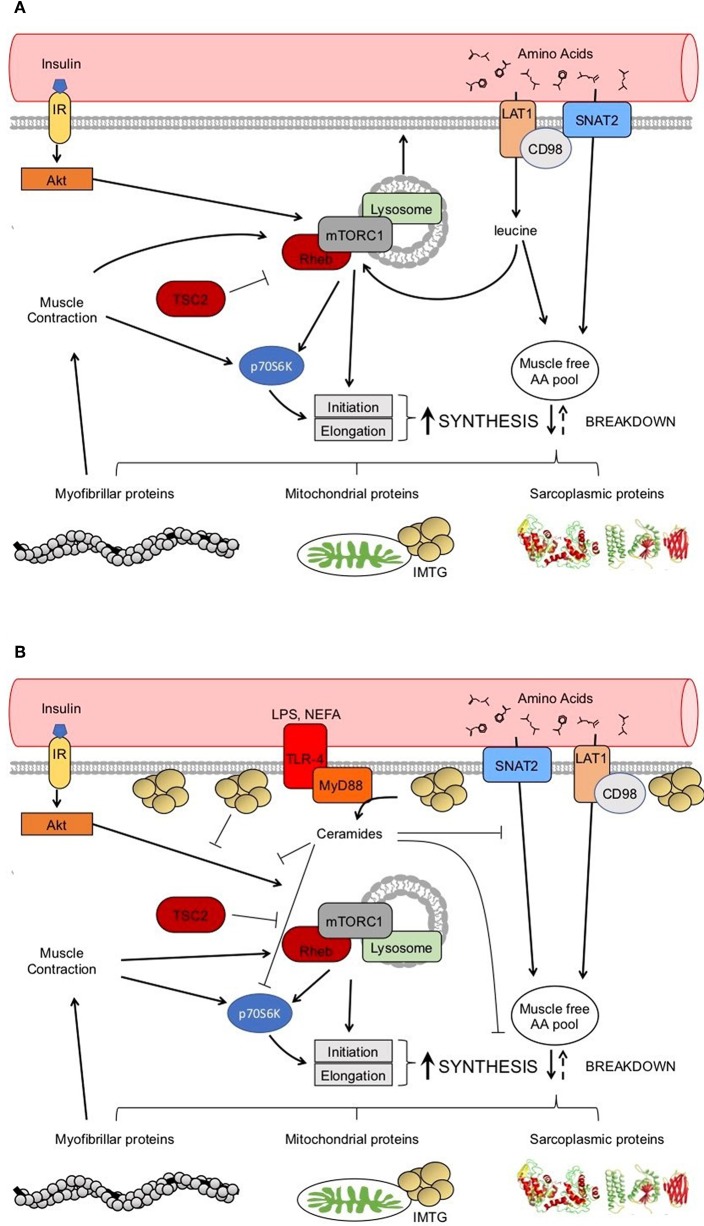
Healthy anabolic signaling in the skeletal muscle **(A)**. Potential dysregulation to anabolic signaling with obesity **(B)**. → indicates a stimulation. ⊥ indicates an inhibition.

Regulation of muscle protein synthesis rates is coordinated by several extra- and intracellular signals, many of which are increased in response to nutrition (e.g., insulin and AAs; Figure 2A) and physical activity (e.g., muscle contraction; Figure 2A). Changes in protein phosphorylation and activity is fundamentally catalyzed by protein-protein interactions, the study of which has led to a greater understanding of and appreciation for the dynamic nature of mRNA translation regulation in human muscle (31). For example, mixed meal ingestion with protein and carbohydrate induces a rise in plasma aminoacidemia and insulinemia that directs the dissociation of Ras homolog-enriched in brain (Rheb) from its negative regulator tuberous sclerosis complex 2 (TSC2) in order to facilitate Rheb association with the mechanistic target of rapamycin (mTOR) to form the mTOR complex 1 (mTORC1) (32, 33). This complex subsequently moves to the lysosome and translocates toward the sarcolemma, which is more proximal to capillaries, AA transporters (e.g., the large neutral amino acid transporter, LAT1 or SLC7A5), and the ribosomal machinery (33–35). This intracellular positioning would presumably be ideally suited to detect and utilize exogenous nutrients for the postprandial muscle protein synthetic response at the level of mRNA translation. In addition, the kinase activity of mTORC1 is essential for the phosphorylation and activation of several proteins involved in ribosomal assembly (e.g., ribosomal protein S6, rpS6; eukaryotic translation initiation factor 4E-binding protein 1, 4EBP1; eukaryotic initiation factor 2, eIF2) either directly, or through downstream kinases (e.g., ribosomal protein S6 kinase 1, p70S6K1) (36, 37). Indeed, phosphorylation of mTOR and these downstream proteins are commonly used readouts of pathway activation (38–42), though it should be appreciated that changes in protein phosphorylation of these candidate markers do not always reflect their kinase activity (43) nor direct proportional changes in muscle protein synthesis (38) in human muscle. Importantly, these processes are also stimulated by muscle contraction (i.e., resistance exercise) with the peripheral targeting of mTORC1 persisting beyond the acute postprandial period (i.e., >3 h) (34), which likely contributes to the sustained p70S6K1 phosphorylation and myofibrillar (i.e., contractile proteins) protein synthetic response with exercise (44, 45). There is also *in vitro* evidence suggesting that, similar to glucose transport, an inducible pool of amino acid transporters (e.g., SNAT2) may also be recruited to the sarcolemma in response to anabolic stimuli, such as insulin, and contribute to the acute regulation of muscle protein synthesis (46). Thus, given the dynamic nature of anabolic signaling events and nutrient transport, any physical or biochemical changes within the skeletal muscle of people with obesity that interferes with these intracellular processes could ultimately contribute to the dysregulation of muscle protein synthesis.

Protein ingestion and exercise have been shown to be two primary anabolic stimuli to human skeletal muscle; protein ingestion being particularly important as it also provides the necessary substrate (i.e., essential AAs) for synthesizing new proteins. The factors involved in the regulation of muscle protein synthesis rates has been studied under a variety of conditions and different populations. From a protein nutrition perspective, a range of 20–40 g of high quality protein is required to ingest in a single meal to maximize the muscle protein synthetic response during the postprandial period in adult men (21, 47–50). Protein type (isolated vs. whole food), exercise pattern (resistance vs. endurance), BMI, and/or age of an individual may all be important factors that modulate the recommended amount of protein to consume in a meal to augment postprandial muscle protein synthesis rates as discussed elsewhere (51, 52).

Muscle protein synthesis, however, is a relatively generic term given the myriad of various structural and regulatory proteins within this tissue. Many studies, however, assess the synthesis of mixed muscle protein, or the entire muscle proteome. In healthy adults, the rates of protein synthesis are not equivalent across sub-fractions of the skeletal muscle (44, 53) and mixed muscle protein synthesis represents a weighted average of the rates of these sub-fractions. However, this approach may obscure important phenotypic differences in rates of protein synthesis occurring in specific protein sub-fractions in response to nutrition or exercise. For example, it has been shown that the stimulation of myofibrillar protein synthesis rates is more sensitive to feeding and exercise during early (i.e., ~5 h) and later (i.e., ~24 h) recovery when compared to the sarcoplasmic protein fraction in healthy adults (44, 54). More importantly, muscle sub-fractional protein synthesis rates are differentially responsive to common stimuli [e.g., insulin, exercise (38–40, 44, 55, 56)] and may be differentially impacted based on the population studied (39, 40, 57). Therefore, to obtain a more complete picture of the potential dysregulation of muscle protein metabolism in clinical populations such as the obese it is advantageous to investigate the response in specific sub-fractions.

## The Impact of Obesity on Muscle Protein Turnover

Basal muscle protein synthesis rates have been assessed in obese individuals in mixed muscle (41, 58) and within the myofibrillar (40, 42, 59–61), sarcoplasmic (40, 42, 61), and mitochondrial (41, 58, 62) protein sub-fractions and generally show no impairment when compared to healthy-weight counterparts. Though, lower basal mixed muscle (41) and mitochondrial (58) protein synthesis rates in individuals with obesity as compared to non-obese individuals have also been reported. The reasons for these discrepant findings are not clear, and may reflect the heterogeneity observed with obesity, which is discussed later on. However, obesity is characterized by several well-known impairments in macronutrient metabolism in skeletal muscle that results in altered regulation of blood glucose and lipids (63). Accumulating evidence also suggests that stimulation of muscle protein synthesis rates during the postprandial period is altered in obese individuals as compared to normal-weight controls (defined as a body mass index; BMI < 25 kg/m^2^) (40–42, 58–60), although this finding is not universal (61, 62). Moreover, the specific alterations to muscle sub-fractions (e.g., mitochondrial, myofibrillar, and/or sarcoplasmic) are also not consistent among studies. These discrepancies between studies may relate to a lack of standardized participant grouping (e.g., healthy-weight (BMI < 25 kg/m^2^) vs. non-obese individuals (BMI < 30 kg/m^2^) as controls) and/or postprandial conditions (i.e., meal ingestion vs. AA infusions) among study designs. A summary of the above studies is presented in Table 1 and their main findings will be discussed in the next sections.

**Table 1:** Effect of obesity on human muscle protein synthesis.

**AMINO ACID INFUSION STUDIES**						
**Experiment**	**Populations studied**	**Conditions**		**Basal MPS OB vs. CON**	**OB Postprandial MPS vs. basal**	**Postprandial MPS OB vs. CON**
Guillet et al. (58)	Young men	Hyper-AA		↓ Mixed	↑ Mixed	↓ Mixed
	OB vs. Non-OB (CON)	hyperinsulinemia		↓ Mito	↔ Mito	↓ Mito
Murton et. al. (59)	Older men	Hyper-AA		↔ Myo	↔ Myo	↓ Myo
	OB vs. HW (CON)	hyperinsulinemia				
Chevalier et al. (61)	Young men	Hyper-AA		↔ Myo	↑ Myo	↔ Myo
	OB vs. HW (CON)	hyperinsulinemia		↔ Sarc	↑ Sarc	↔ Sarc
Tran et al. (41)	Young men	Hyper-AA		↓ Mixed	↑ Mixed	↔ Mixed
	OB vs. HW (CON)			↓ Mito	↑ Mito	↔ Mito
**PROTEIN INGESTION STUDIES**						
**Experiment**	**Populations studied**	**Protein source**		**Basal MPS OB vs. CON**	**OB Postprandial MPS vs. basal**	**Postprandial MPS OB vs. CON**
Beals et al. (42)	Young adults	Lean pork (36 g)		↔ Myo	↔ Myo	↓ Myo
	OB vs. HW (CON)					
Beals et al. (62)	Young adults	Lean pork (36 g)		↔ Mito	↑ Mito	↔ Mito
	OB vs. HW (CON)					
Smeuninx et al. (60)	Older adults	Milk protein isolate (15 g)		↔ Myo	↔ Myo	↓ Myo
	OB vs. HW (CON)					
**EXERCISE STUDIES**						
**Experiment**	**Populations studied**	**Protein source**	**Exercise bout**	**Basal MPS OB vs. CON**	OB Postexercise MPS vs. rest^*^	**Postexercise MPS OB vs. CON**
Hulston et al. (72)	Young adults	N/A	Unilateral knee	N/A	↑ Mixed	↔ Mixed
	OB vs. HW (CON)		extension 4 sets at 70% 1RM			
Beals et al. (40)	Young adults	Lean pork (36 g)	Unilateral knee	↔ Myo	↔ Myo	↓ Myo
	OB vs. HW (CON)		extension 4 sets at 65-70% 1RM	↔ Sarc	↔ Sarc	↔ Sarc

*OB, Obese; CON, control group; HW, healthy-weight; OW, overweight; AA, amino acids; MPS, muscle protein synthesis; Sarc, sarcoplasmic; Mito, mitochondrial; Myo, myofibrillar; Mixed, mixed muscle; ↔, no change/difference; ↑, increased/greater; ↓, decreased/lower*.

* *these studies used a unilateral model—rest leg was used for this comparison*.

Much of the earlier work studying the effects of obesity on the regulation of postprandial muscle protein synthesis rates centered on the intravenous delivery of AAs during a clamp procedure. Under hyperinsulinemic-hyperaminoacidemic clamp conditions, it has been reported that obese men exhibit lower rates of mixed muscle (58), myofibrillar (59), mitochondrial (58) protein synthesis when compared to controls, although evidence is also available that do not support these differences (41, 61). While a hyperinsulinemic-euglycemic clamp may represent the gold-standard for assessing insulin-sensitive glucose disposal, sustained hyperaminoacidemia (with or without hyperinsulinemia) is an atypical stimulus that may be associated with a refractory stimulation of muscle protein synthesis (64). Thus, the physiological relevance of clamp conditions for assessing the impact of protein nutrition on postprandial regulation of muscle protein synthesis is arguably limited.

Relatively few experiments have compared the stimulation of postprandial muscle protein synthesis rates in individuals with obesity vs. healthy-weight individuals under the typical applied setting of ingesting protein-dense foods (42, 60, 62). These experiments, which have incorporated the ingestion of a bolus of high-quality protein [i.e., milk (60) or lean pork (42, 62)], have revealed that myofibrillar (42, 60), but not mitochondrial protein synthesis rates (62), are reduced in people with obesity compared with healthy-weight individuals after the consumption of protein-dense foods. Indeed, protein-dense food ingestion results in a differential pattern of plasma amino acid availability when compared to directly infusing free AAs intravenously. For example, the ingestion of meat, milk and eggs, important sources of protein in many diet patterns, results in peak plasma amino acid availability occurring ~2 h after the meal, which wanes over the latter portion of a postprandial period (65, 66). The same pattern is also witnessed when observing plasma insulin concentrations after protein ingestion in healthy adults (67). By contrast, clamp conditions are on the other end of the continuum and attempt to alter concentrations of AA and/or insulin in a square wave fashion to maintain concentrations at postprandial or supraphysiological levels over an extended period of time, which may have unintended consequences on muscle protein synthesis rates (64). Clamp conditions also bypass the digestive tract, which plays several important roles in stimulating uptake of nutrients from a meal into peripheral tissues (68–70). Collectively, it is clear that the prime anabolic signals (dietary AAs) to muscle behave very differently when provided as a bolus vs. delivered intravenously, and underlines the value of using more practical approaches when characterizing the impact of nutrition on the regulation of muscle protein synthesis rates in a fraction specific manner with health and disease.

## Obesity-Related Anabolic Resistance of Muscle Protein Synthesis

Potential factors underpinning anabolic resistance of muscle protein synthesis with obesity are shown in Figure 2B. Somewhat contradictory to the above data regarding muscle protein synthesis rates, basal phosphorylation of anabolic signaling proteins such as mTOR^S2448^ (40–42), and its downstream target ribosomal protein S6 kinase (p70S6K^T389^) (40, 71), are often quite elevated in people with obesity when compared to normal-weight individuals. These findings are suggestive of greater basal anabolic signaling in the muscle. However, an elevated phosphorylated-state of basal anabolic signaling mechanisms in people with obesity are not universal findings (59, 72), which again serves to reflect the heterogeneity in this condition. Total muscle mTOR protein content is also not always reported (41, 72), and presenting only ratios of phosphorylated: total protein can mask the true levels of phosphorylated mTOR as several studies have reported greater muscle mTOR protein content in individuals with obesity (40, 42). It is currently not fully understood how basal anabolic signals are maintained at these greater levels with obesity. Nevertheless, greater basal mTOR phosphorylation may prevent the characteristic postprandial increase in mTOR phosphorylation in those with obesity (41, 42), perhaps suggesting an upper limit for activation of anabolic signaling through this protein complex. For example, one study showing apparently normal basal mTOR phosphorylation in those with obesity, did show increased phosphorylation of mTOR during the postprandial period (59). Phosphorylation of other target proteins downstream of mTORC1 and/or p70S6K signaling [ribosomal protein S6, rpS6^S240/244^ (58, 90); eukaryotic translation initiation factor 4E-binding protein 1, 4EBP1^T37/46^ (58); eukaryotic initiation factor 2, eIF2^S251^ (44)] are unaltered by obesity indicating that proximal aspects of the anabolic signaling pathway are more disrupted compared to healthy-weight individuals.

## Insulin Resistance and Muscle Anabolism

Resistance to insulin was historically uncommon in the literature, initially only represented by case studies (73). However, by the 1950s it was recognized that a subset of people with type 2 diabetes were less responsive to insulin and this was accompanied with being overweight or obese (74). Given insulin's role in mTORC1 signaling, diminished responsiveness could be an important aspect of muscle protein metabolism during the postprandial period.

Obesity is often associated with elevated basal plasma circulating branched chain AAs (BCAA) levels (75, 76), which may relate to metabolic changes observed in the skeletal muscle of people with obesity (77). Greater plasma BCAAs concentrations are potentially linked to dysregulation of metabolism with obesity, mostly as it relates to insulin resistance (75, 78–81) and chronic activation of the mTORC1 signaling pathway (82, 83). These findings could be relevant to observations of elevated basal mTORC1 signaling in people with obesity (40–42). However, only one study that reported the effects of obesity on the mTORC1 signaling pathway and muscle protein synthesis has shown mild elevations (~20%) in plasma BCAAs in obese vs. lean individuals (61) with the majority reporting no differences (40–42, 58, 60, 84). Collectively this seems to suggest that elevated basal circulating BCAAs may not be significant nor consistent contributors to the dysregulated muscle protein anabolic response in obesity.

Obesity is also associated with elevated plasma non-esterified fatty acids (NEFA) (85). In fact, infusions of lipid with heparin, which increases plasma NEFAs, can blunt insulin sensitivity even in healthy, insulin-sensitive subjects (86). Lipid infusions also impair myofibrillar protein synthesis in healthy participants under hyperinsulinemic-hyperaminoacidemic conditions (87), which suggests a possible role for insulin resistance in the regulation of myofibrillar protein synthesis. A series of experiments examined the relationship between plasma NEFA concentrations and muscle protein synthesis rates in response to protein ingestion in obese participants and healthy-weight controls (42, 62). The participants with obesity in these studies had only subtle differences in postprandial plasma NEFAs, which did not appear to be related to either myofibrillar (42) or mitochondrial protein synthesis (62). In contrast, other have shown that greater intramyocellular lipids are associated with diminished postprandial myofibrillar protein synthesis in obese older adults (60). High intramyocellular lipid is classically associated with insulin resistance in sedentary populations (88), although the causality and mechanistic link for this relationship is not clear. It is worthwhile noting that intramyocellular lipid accumulation in obese human muscle (60) tends to be sub-sarcolemmal as compared to intermyofibrillar in athletic populations (89), which could impair mTORC1 translocation and subsequent downstream activity of this pathway. Alternatively, dysregulated insulin signaling is also attributed to changes in intracellular lipid metabolites (90–92) and in particular the sphingolipid ceramide (92, 93). An *in vitro* study showed that ceramide treatment reduced small neutral amino acid transporter (SNAT2)-mediated sarcolemmal translocation and amino acid transport in L6 myotubes, which translated in an attenuated phosphorylation of p70S6K^T389^ and amino acid induced stimulation of muscle protein synthesis (94). Thus, the relationship between intramyocellular lipid accumulation and/or ceramide production and the intracellular anabolic signaling (e.g., mTORC1) represents a fruitful area for further study.

Efficient delivery of AAs to peripheral tissues is important for the postprandial stimulation of muscle protein synthesis and may be mediated by an insulin-induced vasodilation of the capillary bed (95, 96). Muscle capillarity has been suggested to independently influence peripheral insulin sensitivity and postprandial myofibrillar protein synthesis rates in older adults (97–99), which could implicate a diminished muscle capillary network (100) and/or insulin-induced recruitment (101) in obese individuals as a contributing factor to the anabolic resistance of this population.

Thus, far the impact of obesity *per se* compared to its associated insulin resistance on the dysregulation of postprandial anabolic response is unknown. For instance, overweight young adults with apparently normal insulinemia and homeostatic model assessment of insulin resistance (HOMA-IR) have also been reported to have a blunted myofibrillar protein synthesis response to protein ingestion and greater basal mTOR phosphorylation (42). This could suggest that increased basal mTOR phosphorylation may occur early with weight gain prior to the development of discernible insulin resistance but concomitant with a blunted postprandial anabolic response. Indeed, substantial evidence has mounted that some individuals with obesity remain nearly as insulin sensitive as lean counterparts while other individuals with a similar degree of obesity become insulin resistant (102). Whether differences in insulin sensitivity are predictive of muscle anabolic sensitivity in obese individuals is currently not known.

## Inflammation and Muscle Protein Synthesis

Obesity is associated with chronic low-grade inflammation (103), which has been linked to impaired glucose tolerance (104) and dyslipidemia (105). This includes elevated basal levels of plasma inflammatory biomarkers [i.e., CRP (42, 59, 60, 62, 72), IL-6 (42, 59, 62), TNFα (59)] in obese participants. Because of shared metabolic signaling pathways, skeletal muscle inflammation may also contribute to impaired protein anabolism in obese individuals [Figure 2B (106)]. Indeed, protein metabolism is dramatically altered by high levels of inflammation with trauma or severe illness [e.g., thermal injury (107) or end stage renal disease (108)], which may be related to direct effects of some inflammatory markers (e.g., CRP and TNFα) on suppressing muscle protein synthesis as demonstrated *in vitro* (109, 110).

A few studies have attempted to describe the muscle protein synthetic response to protein ingestion in humans with low-grade inflammation independent of obesity (111, 112). In healthy older men stratified by plasma C-reactive protein (CRP) concentrations, postprandial mixed muscle protein synthetic rates were not different between groups (111). Another group tested the effect of 1 week of ibuprofen or placebo administration on basal and postprandial myofibrillar protein responses to whey protein ingestion in older men with elevated CRP and compared these responses to those of healthy non-inflamed older men (112). In this study, basal and postprandial myofibrillar protein synthesis were not different between ibuprofen and placebo groups. Moreover, both intervention groups (ibuprofen and placebo) had similar basal and postprandial rates of myofibrillar protein synthesis compared with the non-inflamed control group (112). Despite the implication of CRP in attenuating muscle protein anabolism *in vitro* (109), results in humans, and especially obese individuals, are less clear. Nevertheless, elevated basal levels of plasma inflammatory biomarkers in individuals with obesity [i.e., CRP (42, 59, 60, 62, 72), IL-6 (42, 59, 62), TNFα (59)] are associated with basal muscle protein synthesis rates that are indistinguishable from healthy-weight non-inflamed controls, but blunted postprandial myofibrillar protein synthesis rates (42, 59, 60, 62), suggesting the impact of low-grade inflammation (e.g., elevated inflammatory cytokines) may depend on the nutrient environment. Moreover, as discussed above, the muscles of overweight individuals are also anabolic resistant (42), but this group does not show indications of systemic or muscle inflammation (62).

Data concerning the effect of muscle inflammation on postprandial muscle protein synthesis in people with obesity is largely limited to the toll-like receptor 4 (TLR4) signaling pathway. This receptor is involved in innate immunity and is primarily known for responding to endotoxin (113), but is also responsive to NEFA (114) and CRP (115) in circulation. TLR4 signaling involves docking with several intracellular proteins, among these is myeloid differentiation factor 88 (MyD88), which appears to potentiate the intracellular signaling of TLR4-induced insulin resistance (114, 116). Muscle TLR4 protein content correlates with body fat percentage in older adults (117) and is related to NEFA-induced insulin resistance (114). Although, one group found that muscle content of both TLR4 and MyD88 proteins are greater in obese, anabolic resistant adults compared with healthy-weight controls (62), discerning the impact of obesity or low-grade inflammation on differences in the postprandial muscle protein synthetic response is not possible from the experiment described above.

## Exercise to Improve Anabolic Sensitivity With Obesity

Leisure time physical activity is a potent treatment for health and its regular performance reduces mortality (118). Physical inactivity has been linked to numerous adverse health outcomes and is predictive of metabolic health with obesity (119). Physical inactivity has also been linked to anabolic resistance in obese older adults (60) and may contribute to the development of sarcopenic obesity (120). For interventions, exercise represents a structured manner to increase daily physical activity. Exercise training takes many forms, but most can be categorized as either endurance (aerobic) or resistance (strength) exercise, though those lines can be somewhat blurred (e.g., high intensity interval training). Each of these training modalities has differing effects on the muscle phenotype, but the effects on muscle tissue health (e.g., insulin sensitivity, endothelial function) appear to be more universal (121–124). Moreover, as physical activity is an essential component of strategies to improve body composition (125, 126), it is important to consider how the muscle adaptive response is affected by obesity.

Endurance training is commonly recommended to improve health and body composition in individuals with obesity. There is limited data studying the impact of endurance exercise on the muscle protein synthesis rates in individuals with obesity. However, in healthy, but untrained young men, an acute bout of endurance exercise appears to favor the stimulation of mitochondrial, over myofibrillar, muscle protein synthesis rates during the postprandial period, an effect which was not modified by a 10-wk training period (39). The same work also reported that resistance exercise tends to increase myofibrillar muscle protein synthesis, in particular after a period of training (39). These findings may be important for determining exercise prescription in those with obesity, given that myofibrillar protein synthesis seems to more affected by obesity (40, 42, 59–61) than muscle mitochondrial protein synthesis (41, 58, 62).

A single bout of resistance exercise can induce substantial alterations of macronutrient metabolism such as improvements in glucose tolerance (121) and postprandial lipemia (127) in healthy young men. Resistance exercise also potentiates muscle protein synthesis rates compared with feeding alone in healthy-weight young and older men (128), an effect that may persist for up to 2 days (54, 129). It appears that resistance exercise is particularly effective at enhancing the myofibrillar (more so than the sarcoplasmic or mitochondrial) sub-fractional protein synthetic response to protein ingestion in healthy adults (40, 44, 56). Therefore, resistance exercise would ostensibly be an ideal intervention for improving the obesity-related impairment in postprandial myofibrillar protein synthesis rates.

Two studies have assessed the impact of acute resistance exercise on muscle protein synthesis and related anabolic signaling mechanism in people with obesity, the findings of which are summarized in Tables 1, 2 (40, 72). One investigation observed acute resistance exercise increased mixed muscle protein synthesis in the fasted state with no differences in protein synthetic rates nor anabolic signaling molecule phosphorylation (e.g., mTORC1) between obese and healthy-weight adults (72). However, mixed muscle protein synthetic responses represent an average of all muscle proteins, which can have markedly different rates of turnover and contraction and nutrient sensitivities (44, 130, 131). When sub-fractional protein synthetic responses to resistance exercise after protein ingestion are compared, the postprandial myofibrillar protein synthetic response was not further stimulated by resistance exercise in obese vs. healthy-weight groups whereas sarcoplasmic muscle protein synthesis rates were largely unaffected by obesity or exercise (40). We also reported that resistance exercise prior to protein ingestion did not augment phosphorylation of targets downstream of mTORC1 (p70S6K^T389^, 4EBP1^T37/46^) in those with obesity, which contrasted starkly with their healthy-weight counterparts. As highlighted previously, lysosomal targeting of mTORC1, which appears to be mediated by the production of phosphatidic acid (PA) (132–134), is integral to maximize post-exercise myofibrillar synthetic rates in the fed state (135). Interestingly, ceramide has also been reported to blunt PA production in L6 myoblasts (136), which may have contributed to the attenuated myofibrillar protein synthetic response to resistance exercise in obese individuals (40). Nevertheless, these studies collectively underscore the importance of assessing the sub-fractional protein synthetic responses to the independent and combined anabolic effect of resistance exercise and protein ingestion.

**Table 2:** Effect of obesity on muscle anabolic signaling.

**AMINO ACID INFUSION STUDIES**						
**Experiment**	**Populations studied**	**Conditions**		**Basal OB vs. CON**	**OB Postprandial vs. basal**	**Postprandial OB vs. CON**
Murton et al. (59)	Older men	Hyper-AA		↔ mTOR^S2448^	↑ mTOR^S2448^	↔ mTOR^S2448^
	OB vs. HW(CON)	hyperinsulinemia				
Chevalier et al. (61)	Young men	Hyper-AA		↔ p70S6K^T389^	↑ p70S6K^T389^	↓ p70S6K^T389^
	OB vs. HW (CON)	hyperinsulinemia		↔ rpS6^S240/244^	↑ rpS6^S240/244^	↔ rpS6^S240/244^
				↔ 4EBP1^S65^	↑ 4EBP1^S65^	↔ 4EBP1^S65^
Tran et al. (41)	Young men	Hyper-AA		↑ mTOR^S2448^	↔ mTOR^S2448^	↑ mTOR^S2448^
	OB vs. HW (CON)			↔ p70S6K^T389^	↑ p70S6K^T389^	↑ p70S6K^T389^
				↔ eIF2^S51^	↔ eIF2^S51^	↔ eIF2^S51^
Williamson et al. (71)	OB,T2D vs. HW (CON)	Hyperinsulinemia		↔ REDD1	↔ REDD1	↑ REDD1
				↑ p70S6K^T389^	↓ p70S6K^T389^	↔ p70S6K^T389^
				↓ 4EBP1^T37/46^	↔ 4EBP1^T37/46^	↓ 4EBP1^T37/46^
**PROTEIN INGESTION STUDIES**						
**Experiment**	**Populations studied**	**Protein source**		**Basal OB vs. CON**	**OB Postprandial vs. basal**	**Postprandial OB vs. CON**
Beals et al. (42)	Young adults	Lean pork (36 g)		↑ mTOR^S2448^	↔ mTOR^S2448^	↔ mTOR^S2448^
	OB, OW vs. HW (CON)			↔ p70S6K^T389^	↑ p70S6K^T389^	↑ p70S6K^T389^
Gran et al. (84)	Middle-aged men	Dairy protein (31 g)		N/A	↑ mTOR^S2448^	↔ mTOR^S2448^
	OB w/MetS vs. Non-OB (CON)				↔ p70S6K^T389^	↓ p70S6K^T389^
					↑ rpS6^S240/244^	↔ rpS6^S240/244^
		Soy protein (31 g)		N/A	↔ mTOR^S2448^	↔ mTOR^S2448^
					↔ p70S6K^T389^	↓ p70S6K^T389^
					↔ rpS6^S240/244^	↔ rpS6^S240/244^
**EXERCISE STUDIES**						
**Experiment**	**Populations studied**	**Protein source**	**Exercise bout**	**Basal OB vs. CON**	OB Postexercise vs. rest^*^	**Postexercise OB vs. CON**
Hulston et al. (72)	Young adults	N/A	Unilateral knee	↔ mTOR^S2448^	↑ mTOR^S2448^	↔ mTOR^S2448^
	OB vs. HW (CON)		extension 4	↔ p70S6K^T389^	↑ p70S6K^T389^	↔ p70S6K^T389^
			sets at 70% 1RM	↔ 4EBP1^T37/46^	↑ 4EBP1^T37/46^	↔ 4EBP1^T37/46^
Beals et al. (40)	Young adults	Lean pork (36 g)	Unilateral knee	↑ mTOR^S2448^	↔ mTOR^S2448^	↔ mTOR^S2448^
	OB vs. HW (CON)		extension 4 sets	↑ p70S6K^T389^	↔ p70S6K^T389^	↓ p70S6K^T389^
			at 65–70% 1RM	↔ 4EBP1^T37/46^	↔ 4EBP1^T37/46^	↓ 4EBP1^T37/46^

*OB, obese; CON, control group; HW, healthy-weight; OW, overweight; AA, amino acids; mTORC1, mechanistic target of rapamycin; p70S6K, ribosomal protein S6 kinase; rpS6, ribosomal protein S6; 4EBP1, eukaryotic translation initiation factor 4E-binding protein 1; eIF2, eukaryotic initiation factor 2; REDD1, regulated in development and DNA damage 1; MetS, metabolic syndrome; ↔, No change/difference; ↑, increased/greater; ↓, decreased/lower; 1RM, one-repetition maximum strength*.

* *Resting measurement was performed in contralateral non-exercised leg*.

Combined endurance and resistance (i.e., concurrent) exercise training has been demonstrated to have beneficial effects on body composition in adults with overweight or obesity (137). An acute bout of concurrent exercise has been demonstrated to alleviate the suppressive effect of elevated NEFAs on postprandial mixed muscle protein synthesis rates in middle-aged men with overweight or obesity (138). There are relatively few studies that employed a longitudinal design incorporating a combined endurance and resistance exercise approach in obese older adults (139–141). These studies reported rates of mixed muscle protein synthesis before, during, and after exercise with somewhat equivocal results. Two reports showed that increased multi-modality physical activity (endurance + resistance) over a 3 month period increases basal mixed muscle protein synthesis rates, but the magnitude of postprandial stimulation of muscle protein synthesis rates was not affected (140). In contrast, the same group also reported, in a similar population, that weight loss over 12 months of caloric restriction with multi-modality physical activity does not change either basal or postprandial mixed muscle protein synthesis rates (141). That study did show that during active weight loss, measured at 3 months of the intervention, the postprandial mixed muscle protein synthesis rates were substantially elevated. The latter finding indicates that prolonged energy restriction with relatively low protein intake (1.0 g/kg/day) may hamper the muscle anabolic response to multi-modality exercise (141).

Several studies have shown that greater dietary protein (>1.2 g/kg/day) helps to preserve muscle protein synthesis rates during caloric restriction-induced weight loss (~40% energy restriction) in healthy-weight (142) and individuals with overweight or obesity (10, 143). In overweight and obese men, the amount of dietary protein required to sustain muscle protein synthesis rates during caloric restriction could be even greater when a high volume of exercise (resistance training and high-intensity intervals) is also performed (143). These studies (10, 141, 143) serve to underscore the importance of considering both nutrition and physical activity when designing interventions to treat obesity and/or its co-morbidities.

## Conclusions

Protein ingestion is an important component of a healthy diet and has been touted for its potential to facilitate weight loss for those with obesity (144). When studies are considered together, obesity primarily affects the postprandial myofibrillar protein synthetic response to nutrition and exercise (Table 1), which is likely related to altered intramyocellular signaling cascades (Table 2). Identification of mechanisms responsible for greater basal anabolic signaling molecule phosphorylation could potentially yield novel therapeutic targets.

Obesity is an inherently variable condition, which likely explains the observations discussed throughout this review. For example, insulin resistance can manifest itself as impaired fasting glucose, glucose intolerance, or both (145). In fact, the variability in glucose metabolic outcomes with obesity has been extensively discussed (102, 145). With this in mind, differences in insulin sensitivity or inflammation may impact muscle protein synthetic responses and explain some of the variability observed in the various studies discussed in this review.

The studies of acute resistance exercise discussed above employed robust exercise protocols in excess of most recommendations for untrained weightlifters (146–148). It is remarkable that this exercise prescription was insufficient to augment myofibrillar protein synthesis rates after protein ingestion. There is potential that increasing the exercise volume could have a positive impact on postprandial myofibrillar protein synthesis rates; similar to improvements seen in older anabolic resistant adults (149). Future studies should focus on long-term interventions that include combined diet and exercise strategies to reduce obesity and examine the impact of weight loss and/or exercise training status on postprandial muscle protein synthesis and anabolic signaling. These long-term studies could also benefit from the use of deuterium oxide (heavy water) to determine free-living rates of muscle protein synthesis during an intervention period. However, differences between nutritional interventions (e.g., protein type, leucine dose) on acute rates of myofibrillar protein synthesis in response to a single meal ingestion with traditional primed constant infusions may be less pronounced when assessed by D2O in a free-living environment (150, 151), highlighting the need for additional research utilizing a variety of stable isotope methodologies to study the presence and consequence of obesity-related anabolic resistance. Dietary interventions should focus on ensuring adequate protein nutrition (~1.2 g/kg/day). Ideally, future studies would incorporate more comprehensive metabolic profiling (e.g., measures of insulin sensitivity) that would allow better insight as to how the phenomena of metabolic (ab)normality with obesity (102) affects muscle protein synthesis responses to dietary protein and exercise.

## Author Contributions

JB wrote the first draft of the manuscript. NB, DM, and SV critically revised the text and made substantial contributions to the manuscript. All authors approved the final version of the manuscript.

### Conflict of Interest Statement

The authors declare that the research was conducted in the absence of any commercial or financial relationships that could be construed as a potential conflict of interest.
